# Using qPCR to compare the detection of *Plasmodium vivax* oocysts and sporozoites in *Anopheles farauti* mosquitoes between two DNA extraction methods

**DOI:** 10.3389/fpara.2023.1063452

**Published:** 2023-03-16

**Authors:** Lincoln Timinao, Esther W. Jamea, Michelle Katusele, Thomas R. Burkot, Stephan Karl

**Affiliations:** ^1^ Papua New Guinea Institute of Medical Research, Madang, Papua New Guinea; ^2^ Australian Institute of Tropical Health and Medicine, James Cook University, Smithfield, QLD, Australia

**Keywords:** *Anopheles farauti*, oocysts, sporozoites, *Plasmodium vivax*, Papua New Guinea

## Abstract

**Background:**

Currently, the gold standard to assess parasite developmental stages in mosquitoes is light microscopy. Microscopy can miss low-density infections, is time-consuming and not species-specific. Enzyme-linked immunosorbent assay (ELISA) has been the alternative technique to evaluate the infectivity of mosquitoes especially in field studies however it is semi-quantitative. Molecular techniques that have been used to detect the mosquito stages of malaria parasites including *P. vivax*. Here, we present a quantitative real-time assay (qPCR) that can be used to detect low-density *P. vivax* oocyst and sporozoite infections while comparing parasites extracted by the conventional DNA extraction and heating methods.

**Methods:**

Colony reared *Anopheles farauti* mosquitoes were exposed to blood samples collected from infected individuals using a direct membrane feeding assay. The fully fed mosquitoes were kept for 7 and 14 days post-feed before dissection to confirm presence of oocysts and sporozoites. Infected mosquito guts and the salivary glands (with the head and thorax) were stored and DNA was extracted either by heating or by performing conventional column-based DNA extraction. Following DNA extraction the infected samples were subjected to qPCR to detect *P. vivax* parasites.

**Results:**

DNA extraction of 1 or more oocysts by heating resulted in an overall sensitivity of 78% (57/73) and single oocysts infections were detected with a sensitivity of 82% (15/17) in the heating arm. We observed a 60% (18/30) sensitivity with sporozoites where DNA was extracted using the conventional DNA extraction method. We show that the heating method significantly improved the detection of oocysts over conventional DNA extraction. There was no significant difference in the DNA copy numbers when comparing the detection of oocysts from the conventional DNA extraction versus heating. However, we observed that the DNA copy numbers of the sporozoites detected in the heating arm was significantly higher than in the conventional DNA extraction arm.

**Conclusion:**

We have adapted a qPCR assay which, when coupled with heating to release DNA reduces sample processing time and cost. Direct qPCR after heating will be a useful tool when investigating transmission blocking vaccines or antimalarials or when evaluating field caught mosquitoes for the presence of malaria parasites.

## Background

Malaria is a significant health problem in 85 countries and nearly half of the world’s population is living in areas with risk of malaria transmission (World Health Organization, 2021). Despite the efforts to curb malaria globally, it has proven difficult to achieve a steady decrease in malaria cases over the years, highlighting the need for additional interventions. Transmission blocking interventions such as vaccines and antimalarials can be effective tools used to prevent the spread of malaria parasites (Blagborough et al., 2013).

Human-to-Mosquito transmission, and the activity of potential transmission-blocking compounds, can be investigated using artificial systems such as membrane feeding set ups. Membrane feeding assays (MFAs) were initially developed by Rutledge and others in the 1960s (Rutledge et al., 1964). In MFAs malaria parasites (whether cultured *in vitro* in the laboratory or from infected patients) are fed to the mosquitoes (Miura et al., 2013; Miura et al., 2020). Transmission success can be evaluated by the observation of various parasite developmental stages in the mosquito in particular, the oocysts in the midgut and sporozoites in the salivary glands using light microscopy. Traditionally, light microscopy (LM) was used for assessing the presence or absence of the oocysts or sporozoites in the mosquito however, there are inherent limitations with LM detection of parasite mosquito stages. These include labor intensiveness, the requirement for trained personnel and the resulting low throughput. In addition, low-level infections can easily be missed or misdiagnosed, and the differentiation between parasite species in co-endemic settings is not possible.

MFAs can be operationally challenging particularly in resource-limited settings. Since there is no continuous *P. vivax* culture, access to infected individuals is currently the only option (Bermúdez et al., 2018). This comes with inherent issues, including in some instances the lack of correlation between the gametocyte densities in natural infections and either the oocyst density or the frequency of mosquito infection (Sattabongkot et al., 1991; Schneider et al., 2007). In order to study transmission of malaria parasites derived from infected individuals, a high-throughput method to detect oocysts and sporozoites with high sensitivity is beneficial.

To overcome the limitations of microscopy a number of assays have been developed to enable high throughput detection of parasites in the mosquito gut and salivary glands. These assays include ELISA to detect the circumsporozoite protein (CSP) in mosquito lysates (CSP-ELISA) (Beier et al., 1987; Kumpitak et al., 2021; Sutcliffe et al., 2021), bioluminescence assays to detect transgenic parasites with the green fluorescence protein (GFP) (Delves and Sinden, 2010; Stone et al., 2014; Singer and Frischknecht, 2021), near-infrared spectroscopy (NIRS) to detect parasites within mosquitoes (Maia et al., 2019; Da et al., 2021), enhanced chemiluminescent slot blot (ECL-SB) for detecting *Pf*CSP in mosquito samples (Kumar et al., 2014; Grabias et al., 2017) and molecular detection of *Plasmodium* DNA (Boissière et al., 2013; Marie et al., 2013; Sazed et al., 2021). Although the CSP-ELISA is relatively robust and cost effective it is only semi quantitative (Beier et al., 1987; Kumpitak et al., 2021; Sutcliffe et al., 2021). An assay that is quantitative will enable us to know density of the malaria parasites in the mosquito infection. Bioluminescence GFP assays allow for high-through-put but it cannot be used with wild parasites (Delves and Sinden, 2010; Stone et al., 2014; Singer and Frischknecht, 2021). NIRS has been successfully used to detect *P. falciparum* parasites in lab reared mosquitoes with relatively high accuracy but it is still semi quantitative (Maia et al., 2019; Da et al., 2021). ECL-SB assays can potentially be used to screen large numbers of mosquitoes for oocysts with high sensitivity and specificity (Kumar et al., 2014; Grabias et al., 2017). However, this assay is not quantitative. Various qPCR-based methods have been successfully developed and used to detect blood stage and mosquito infection. However, some qPCR are still semi quantitative mainly due to the design of the qPCR where nonspecific SYBR-green or EVA-green fluorescent dyes were used (Boissière et al., 2013; Marie et al., 2013; Chaumeau et al., 2016; Sazed et al., 2021). Taqman assays are an alternative to SYBR-based real time assays. Taqman assays utilize hydrolysis probes that bind to the target sequence and provides a means to quantify the parasite DNA. The Taqman hydrolysis probes have been used to detect blood stage parasites by targeting the 18S ribosomal RNA gene (Rockett et al., 2011; Wampfler et al., 2013; Wang et al., 2018). Taqman assays are able to detect parasites at levels 4-5 fold lower than expert thick film microscopy (Malhotra et al., 2005; Rantala et al., 2010). Taqman assays detect *P. falciparum* (Bass et al., 2008; Marie et al., 2013; Graumans et al., 2017; Wang et al., 2018) and *P. vivax* parasites in mosquitos using minor grove binding (MGB) probes (Bass et al., 2008; Rao et al., 2009; Bickersmith et al., 2015; Graumans et al., 2017). Minor groove binding probes increase the specificity of the probe binding to the target DNA sequence as compared to unmodified probes and limits cross-hybridization of primers and probes in duplexes (Kutyavin et al., 2000).

Bass and colleagues established a qPCR assay where they evaluated field caught mosquitoes for the presence of *P. vivax* sporozoites in the head and thorax of individual mosquitoes. They did not investigate the qPCR detection of oocysts or the intensity of sporozoite infections (Bass et al., 2008). Rao and colleagues established a multiplex qPCR to detect *Wuchereria bancrofti, P. falciparum*, and *P. vivax* in pools up to 23 field caught mosquitos but did not distinguish between potential sporozoite or oocyst infections (Rao et al., 2009). Bickersmith and colleagues also established a qPCR assay on individual field caught mosquitoes but did not distinguish between the oocyst and sporozoite stages as it was not part of the study design (Bickersmith et al., 2015). Graumans and colleagues also established a qPCR assay where they successfully detected *P. vivax* oocysts stages in mosquitoes but did not investigate the detection of a single *P. vivax* oocyst as it was not part of the study design (Graumans et al., 2017). Also they did not investigate the qPCR detection of *P. vivax* sporozoites.

Sample processing time is an important aspect to consider when setting up an MFA or when processing field collected samples. This includes extracting DNA through to qPCR detection of the parasites in the mosquito. DNA extraction using commercially available kits can usually takes several hours depending on the number of samples that are being processed. In a study by Bass and colleagues they heated the mosquito samples for 10 minutes at 95°C and directly performed qPCR after thus reducing the sample processing time (Bass et al., 2008). However, they did not evaluate the heating technique against the conventional DNA extraction method. This study addresses the key knowledge gap that exists in setting up a sensitive Taqman qPCR assay for both oocysts and sporozoites with known infection densities and compare the mosquito preparation methods of conventional DNA extraction versus heating.

## Methods

### Mosquito rearing

*Anopheles farauti* mosquitoes were reared at 28 ± 8 °C and 68 ± 25% relative humidity (RH) on an 11 h dark and 12 h light including a 30 min dusk and 30 min dawn period. The larvae were fed ground fish food (Marine Master, Tropical Fish Flake) while the adults were provided with 10% sucrose (Ramu Sugar) solution available as soaked cotton wool balls placed on top of the mosquito cages as previously described (Timinao et al., 2021b). Individuals who provided informed consent performed direct skin feeding to maintain our colony mosquitoes.

### Sample collection

This study was conducted at the Papua New Guinea Institute of Medical Research (PNGIMR). Ethical approval was received from the PNG Medical Research Advisory Committee (MRAC #16.01). Patients at Yagaum Clinic in Madang Province of PNG, who consented to participate in the study were recruited. Patients were tested with malaria rapid diagnostic tests (RDTs). In the current study the CareStart Malaria Pf/PAN (HRP2/pLDH) Ag Combo RDT kits (Access Bio, Cat No. RMRM-02571CB) were used. Thick and thin blood films were prepared according to WHO methods for evaluation by a certified microscopist. The blood slides were then stained for 30 minutes using 4% Giemsa (Sigma-Aldrich, Australia) stain (World Health Organization, 2010). Slides were read by the microscopist to identify the presence of the parasites, the species and stages of the parasite in the blood. Parasite density was calculated using the assumption that one microliter of blood contains 8000 white blood cells (WBC) (World Health Organization, 2010) Venous blood samples (5-6mL) were collected from microscopy positive patients in BD Vacutainer ^®^ sampling tubes coated with lithium heparin (BD, Australia). Hemoglobin was measured using a HemoCue^®^ hemoglobin analyzer (HemoCue, Australia). Axillary temperature was taken using a digital thermometer and weight was measured with a bathroom scale (precision ±0.1g). After collection of the blood sample, the BD Vacutainer ^®^ was then immediately stored in a beverage cooler flask (Coleman Company Inc, USA) filled with water adjusted to a temperature of 38°C. A digital thermometer was used to monitor the temperature of the cooler flask. The blood sample was then transported to the insectary for membrane feeding. Transportation time between health facility and laboratory was around 10 minutes.

### Direct membrane feeding assay

At the insectary 3-5 days-old *Anopheles farauti* colony mosquitoes were prepared the previous day and dry starved (i.e., without any sugar or water) overnight. A total of 2 paper cups of 50 mosquitoes per cup were prepared for each feed. Baudruche membrane (Wilco Biotech, USA) was used to feed the mosquitoes through a water-jacketed glass feeder as described previously (Timinao et al., 2021b). Once a blood sample arrived at the insectary it was immediately fed to the mosquitoes for 20 minutes. Unfed mosquitoes were removed and only the fully fed mosquitoes were kept until day 7 post feed when one cup was dissected for oocysts as previously described (Timinao et al., 2021a). The dissected mosquito guts with oocysts were then stored in phosphate-buffered saline (pH~7.4) solution (PBS) in 2mL Eppendorf tubes at -20°C and then the samples were selected for the thermal treatment and DNA extraction arms. The total number of mosquitoes with single oocyst infections together with those with more than one oocyst per mosquito were down-selected for DNA extraction and heating (**Tables S1, Supplementary 1**). The second cup was held until day 14 post feed for detection of sporozoites. The dissections of salivary glands were done by trained microscopists. The dissection for salivary glands were done in a pool of PBS solution. Once the salivary glands were removed from the thorax they were placed on a microscopy slide with a cover slip placed on top. The salivary glands were then taken and viewed under a microscope at 40X magnification to identify the presence of sporozoites. An estimation of the sporozoite infection was made by classifying them into the following categories; low (1-20 sporozoites), moderate (21-100) and high (>100). The salivary glands that were infected with sporozoites were then carefully transferred from the slides to 2mL Eppendorf tubes and then stored in 100 - 200µL of PBS solution together with the head and thorax. The stored salivary glands were then split between the DNA extraction and the heating method (**Tables S2, Supplementary 1**).The salivary glands that were infected with sporozoites were then carefully transferred from the slides to 2mL Eppendorf tubes and then stored in 100 - 200µL of PBS solution together with the head and thorax.

### Heating and DNA extraction

Parasite DNA was extracted using two methods, the conventional DNA extraction with a commercial kit and heating. In this study we used the FavorPrep^®^ DNA extraction kits (Favorgen Biotech Corp, Ping Tung, Taiwan) and performed DNA extraction according to the protocol for extraction of genomic DNA from tissues and for red blood cells and the DNA was eluted in a final volume of 50 µL of elution buffer. The mosquito samples were taken out of the freezer and allowed to defrost on the bench. The samples were vortexed for 30 seconds and then centrifuged for 10 seconds prior to DNA extraction. In the heating method, the down-selected samples with oocyst/s and sporozoites were then vortexed for 30 s and then centrifuged for 10 s at 500 g and then heated at 99°C for 10 min and then cooled on ice prior to performing qPCR (Bass et al., 2008).

### Quantitative real time PCR (qPCR)

Following heating and DNA extraction of the samples, an established Taqman qPCR assay that utilizes MGB probes was performed to quantify the infection and determine the parasite species (Wampfler et al., 2013). This Taqman qPCR assay was used to detect blood stage parasites. Briefly, this qPCR assay targets the conserved region of the 18SrRNA gene for both *P. falciparum* and *P. vivax*. The quantification of parasite copy numbers is derived from synthetic plasmid DNA of known concentrations that are included in each run.

The plasmid concentrations are as follows; 10,10^2^,10^3^ and 10^4^ copies. The plasmid concentrations of 10-10^3^ are run in duplicates. The Cq values (number of cycles that were needed for the fluorescence signal to reach a quantification threshold) of the plasmids of known concentrations are then plotted on a graph against the log starting quantity. A line of best fit (standard curve) is then constructed. The Cq values of the samples are then used to derive the starting quantity from the line of best fit. **Figure S1** in **Supplementary 2** illustrates this.

The qPCR was performed on a CFX96 Touch Real-Time Detection System (Bio-Rad, Australia). The primer and probe sequences together with the reaction mix and the thermo profile are shown in **Table S1**–**S4** in the **Supplementary 3** document.

### Statistical analysis

Statistical analysis was performed using GraphPad Prism (ver. 8.0) and Stata 13 (StataCorp, College Station, TX, USA). The Mann-Whitney test was used to compare the DNA copy numbers between the heating and DNA extraction of mosquito guts with known oocysts counts. The Mann Whitney test was also used to compare the DNA copy numbers between the DNA extraction and the heating method for the sporozoites. The two sample test of proportions was used to compare the proportions of microscopy positive samples that were confirmed by qPCR in the heating and DNA extraction arms.

## Results

A total of 68 patients were recruited (**Table 1**).

**Table 1:** Demographic and clinical data for the study population.

Demography	Median or n/N	IQR (Q1,Q3)	Range	%
Age in years (n=68)	14	11 (10, 21)	4-56	
Female (n=68)	24/68			35.3
Weight, kg, (n=68)	40.5	30 (25, 77)	15 - 77	
Hemoglobin, g/dl, (n=65)^*^	9.9	2.9 (8.6, 11.5)	5.6 - 15.5	
Temperature, ˚C, (n=68)	36.5	0.725 (36.2, 36.9)	35.2 - 39.9	
Fever, >37.5 ˚C, (n=68)	9/68			13.2

^*^Data were not collected for all 68 patients.

**Table 2** shows the results from the three diagnostic methods used.

**Table 2:** Diagnostic results by RDT, microscopy and qPCR.

Diagnosis		n	n/N (%)	95% CI
**RDT**	HRP2	1	1/68 (1.5)	0.04 - 7.9
	pLDH	60	60/68 (88.2)	78.1 - 95.8
	HRP2 & pLDH	7	7/68 (10.3)	4.2 - 20.1
Microscopy^*^	*P. falciparum* asexual with gametocytes	5	5/68 (7.4)	2.4 - 16.3
	*P. vivax* asexual only	10	10/68 (14.7)	7.3 - 25.4
	*P. vivax* asexual with gametocytes	43	43/68 (63.2)	50.7 - 74.3
	*P.falciparum* & *P.vivax*	7	7/68 (10.3)	4.2 - 20.1
	Microscopy negative	3	3/68 (4.4)	0.9 - 12.4
**qPCR** ^§^	*P. falciparum*	4	4/68 (5.9)	1.6 - 14.4
	*P. vivax*	43	43/68 (63.2)	50.7 - 74.6
	PCR negative	21	21/68 (30.9)	20.2 - 43.3

^*^We did not detect any infection with P. falciparum asexual by microscopy or ^§^both P. falciparum and P. vivax by qPCR.

The number of positive samples per test is n. The total number of samples is N=68. Population averages (n/N (%)) and 95% confidence intervals of proportions (95% CI) are also provided.

**Figure 1:**
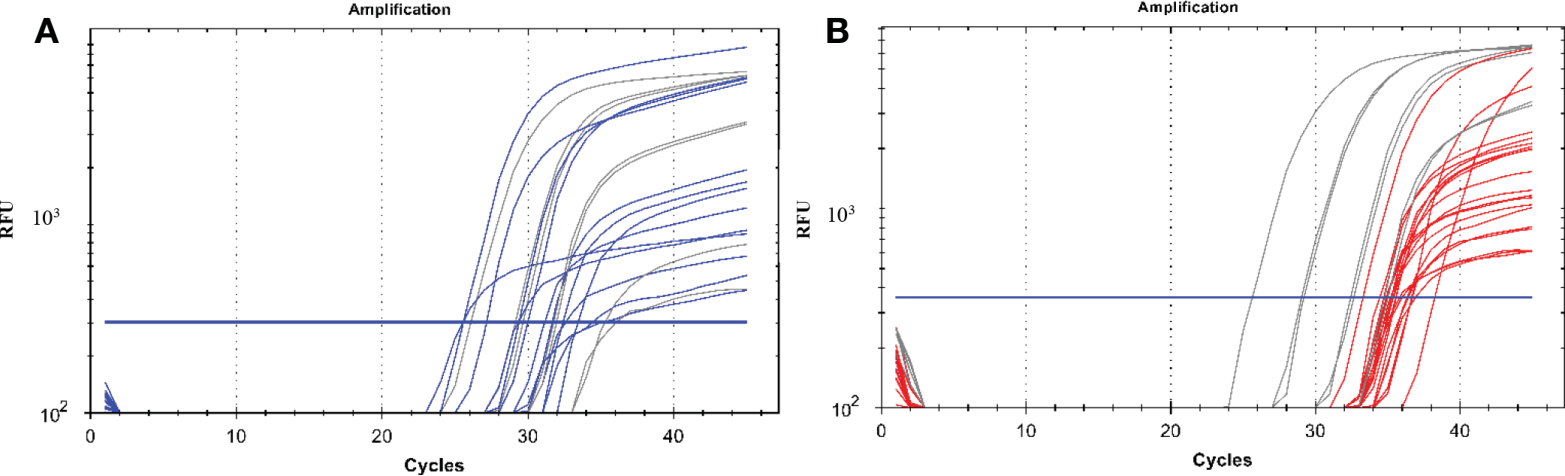
A qPCR amplification plot showing successful amplification of malaria parasite DNA from oocysts and sporozoites. **(A)** represents the amplification of parasite DNA from oocysts with the blue curves being the parasite DNA from mosquito gut samples and the grey lines representing DNA from plasmids of known concentrations which were used as positive controls starting from 10, 10^2^, 10^3^ and 10^4^ copies. **(B)** represents the amplification of parasite DNA from sporozoites from the mosquito salivary glands with the red curves being the parasite DNA while the grey lines representing DNA from plasmids of known concentrations which were used as positive controls starting from 10, 10^2^, 10^3^ and 10^4^ copies. The blue horizontal line represents the threshold value; any curve above this is considered an infection. *RFU, relative fluorescence unit*.

We detected *P. vivax* oocyst and sporozoite stages of the malaria parasites in the mosquitoes using our established protocol. **Figure 1** shows exemplary amplification curves from a qPCR run.

A total of 73 and 72 mosquito samples had at least one oocyst in the mosquito gut which was detected by microscopy for the heating and DNA extraction arms respectively. We observed a significantly higher proportion of mosquito samples that were confirmed by qPCR in the heating arm 78% (57/73) as compared to the DNA extraction arm, 39% (28/72) (p<0.0001).

A total of 17 mosquitoes with single oocysts according to microscopy were processed in both the heating and the DNA extraction arm (**Table 3**). We observed a statistically significant difference with the detection of oocysts by qPCR between the heating arm with a sensitivity of 82% (15/17) and the DNA extraction arm with a sensitivity of 29% (5/17) (p=0.0019).

**Table 3:** Comparison of microscopy positive and qPCR positive oocysts.

	Microscopy		qPCR		
	No. of mosquitoes	No. of oocysts/mosquito	No. of mosquitoes	Sensitivity %, (n/N)	95% CI
**Heating**	17	1	15	88 (15/17)	63 - 99
	20	2 - 10	14	70 (14/20)	46 - 88
	36^*^	> 1	33	92 (33/36)	78 - 98
**Total**	73	>1	62	85 (62/73)	75 - 92
**DNA Extraction**	17	1	5	29 (5/17)	10 - 65
	16	2 - 10	6	38 (6/16)	15 - 65
	39^*^	> 1	18	46 (18/39)	30 - 63
**Total**	72	>1	29	40 (29/72)	29 - 53

^*^This represents 36 pools of midguts with oocysts and not 36 mosquitoes.

**Figure 2:**
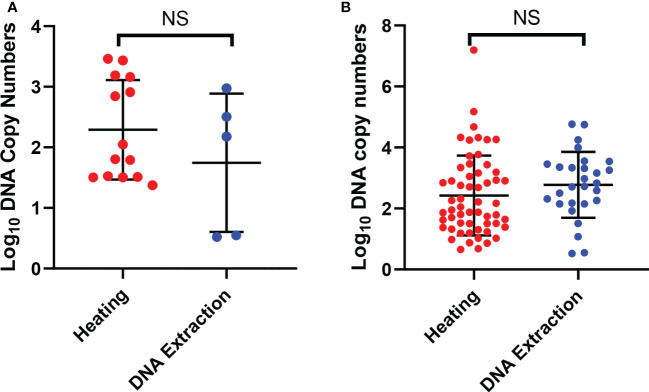
Detection of oocysts using qPCR. **(A)** shows the DNA copy numbers from the single oocysts that were detected by qPCR in the two arms. **(B)** shows DNA copy numbers of all the mosquito samples with one or more oocysts that were detected by qPCR in the two arms. The error bars show the mean and the standard deviation. The dots are mosquitoes. *NS, Not significant*.

When comparing only the oocysts that were successfully detected by qPCR we observed no significant difference between the copy numbers when comparing the detection of parasites from both arms for single oocysts. The observed mean of the log10 transformed copy number data was 2.3 (SD, ± 0.82) for the heating and 1.7 (SD, ± 1.1) for the conventional DNA extraction (**Figure 2A**). Also there was no significant difference in the DNA copy numbers between the two arms with all mosquitoes with oocysts. We observed that the log10 transformed copy number data mean was 2.4 (SD, ± 1.3) for the heating and 2.8 (SD, ± 1.1) for the conventional DNA extraction (**Figure 2B**). We also did not observe any correlation with the DNA copy numbers and the oocyst numbers (**Figure 2B**). We also did not observe any correlation with the DNA copy numbers and the oocyst numbers.

A total of 60 mosquito samples positive for sporozoites by microscopy underwent heating (n=30) and DNA extraction (n=30) (**Table 4**). We observed no significant difference with the detection of sporozoites by qPCR between the heating arm with a sensitivity of 40% (12/30) and the DNA extraction arm with a sensitivity of 60% (18/30) (p=0.121).

**Table 4:** Comparison of microscopy positive and qPCR positive sporozoites.

	Microscopy		qPCR	
	Sporozoite classification^*^	No. of mosquitoes	No. of mosquitoes	Sensitivity%,(n/N)
**Heating**	High	17	4	23.5 (4/17)
	Moderate	9	5	55.6 (5/9)
	Low	4	3	75 (3/4)
**Total**		30	12	40 (12/30)
**DNA Extraction**	High	17	13	76.5 (13/17)
	Moderate	9	4	44.4 (4/9)
	Low	4	1	25 (1/4)
**Total**		30	18	60 (18/30)

^*^ Sporozoite classification: High - >100 sporozoites, Moderate - 21-100 sporozoites, Low - 1-20 sporozoites.

**Figure 3:**
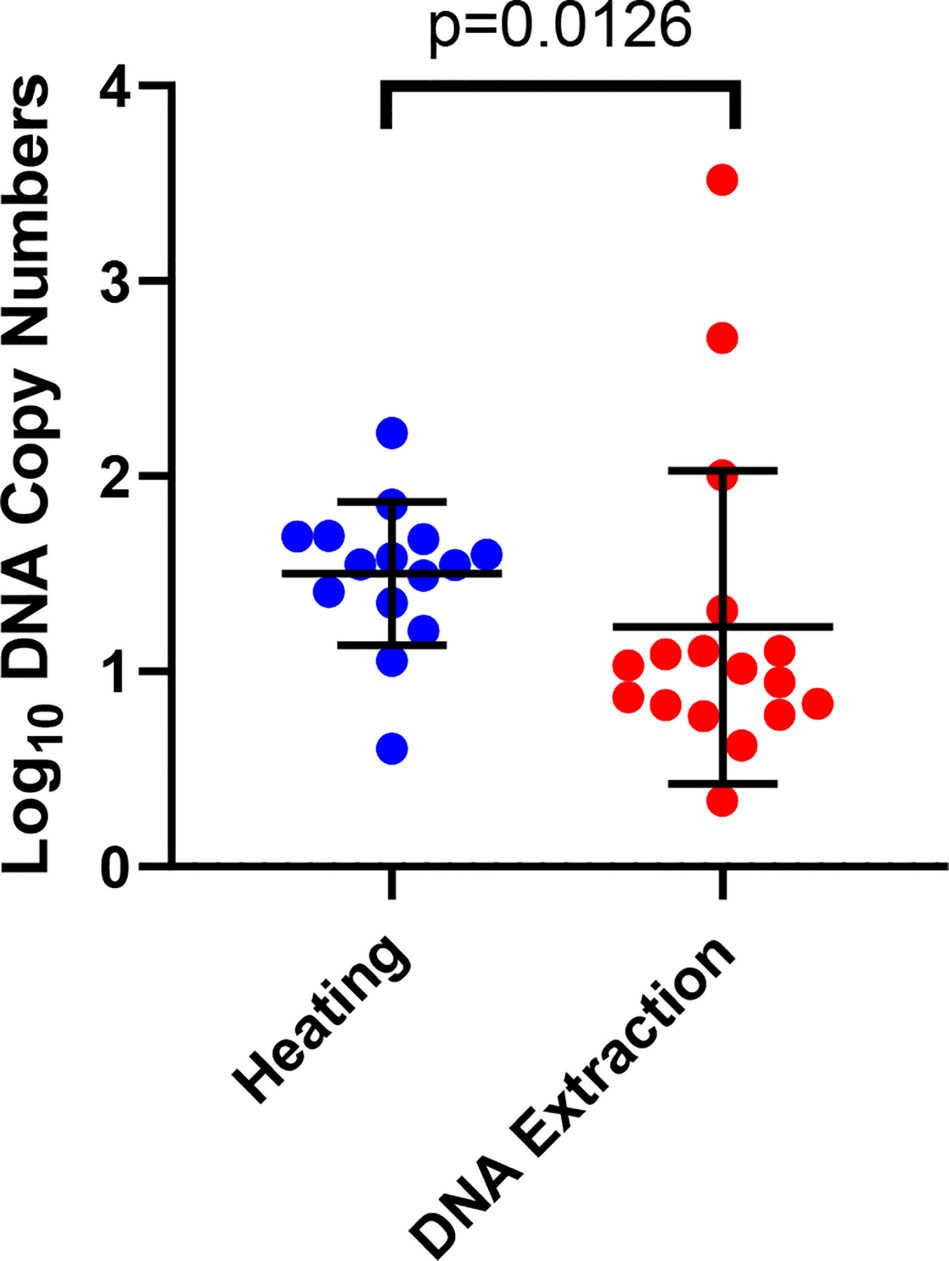
Detection of sporozoites by qPCR in the heating and DNA extraction arms. The error bars show the mean and the standard deviation. Each dot represents a mosquito.

We observed significantly higher DNA copy numbers (p=0.0126) in the qPCR detection of sporozoites in the heating arm as compared to the DNA extraction arm (**Figure 3**). We noted that there was a gradual increase in the mean DNA copy number from Low to High sporozoite count (Low: 12.78 (SD, ± 19.38), Moderate: 29.85 (SD, ± 28.08) and high: 187.29 (SD, ± 772.95).

## Discussion

This study describes the adaptation of a high-through-put qPCR based technique for detecting low levels of oocysts and sporozoites and the evaluation of the conventional DNA extraction method versus heating. The qPCR assay is sensitive enough to detect midgut infections with single oocysts. Furthermore, this assay was able to detect low sporozoite infections by microscopy.

Here we have established a qPCR assay that utilizes the Taqman hydrolysis MGB-probe with increased sensitivity in detecting the *P. vivax* parasite target gene and can potentially enable increased through-put for large scale transmission studies. A number of studies have validated TaqMan qPCR assays for detecting *P. vivax* oocysts and/or sporozoites (Bass et al., 2008; Rao et al., 2009; Bickersmith et al., 2015; Graumans et al., 2017) but have not investigated the limit of detection. We have shown that this qPCR assay is sensitive in detecting low *P. vivax* oocyst and sporozoites infections in mosquitoes.

We show that there is a higher chance of detecting single oocyst infections when heating the dissected midgut compared to the common method of performing DNA extraction. We also show that there is no significant difference between the detection of the parasite’s DNA copy numbers between heating and DNA extraction especially with low infections indicating that heating has a similar DNA output as the common DNA extraction method. We further observe that there is no significant difference between the DNA copy numbers between the two arms with one or more oocysts.

The current study revealed no significant difference in the qPCR detection of sporozoites between the two techniques used to extract DNA from the microscopy positive salivary glands together with the head and thorax. However, heating yielded significantly higher quantities of DNA copies demonstrating the superior performance of heating over the DNA extraction method.

We also observed a higher qPCR detection rate of positive samples with oocysts than with sporozoite samples and this may be due to sporozoites being lost during transferring from glass slides to tubes for storage. Furthermore, the polyploid nature of the oocysts may have contributed to a higher detection of oocysts as compared to sporozoites.

To our knowledge, this is the first research evaluating heating of mosquito guts and salivary gland (with head and thorax). We show that heating is the better option for releasing oocyst and sporozoite DNA and significantly reduces sample processing time and ensures that samples are processed with high efficiency. It also reduces the cost of processing a sample by skipping DNA extraction step using a conventional DNA extraction kit. Bass and colleagues did use heat to free their *P. falciparum* sporozoite DNA prior to performing qPCR but did not evaluate the sensitivity of the technique (Bass et al., 2008). Although similar studies have not been done on mosquitoes, we found that similar comparisons were made with bacteria where they evaluated heating the samples versus using commercially available DNA extraction kits. They found no significant difference between the PCR output from both techniques and suggested that heating was efficient, simple, cheap and suitable for high-through-put (Dashti et al., 2009; Dimitrakopoulou et al., 2020). Similar to what was seen in the case of bacteria, heating the mosquito midguts and salivary glands yielded similar qPCR detection rates for sporozoites while higher detection rates with oocysts as compared to DNA extraction.

Using the heating method will greatly reduce the time taken to process the samples. It takes almost 2 hours to process a single sample from the mosquito stage to a DNA sample before qPCR can be performed on the DNA aliquot. It would take less than 20 minutes to process a single sample when heating the mosquito sample prior to performing qPCR.

There are limitations to the present study. Other studies which used the same qPCR protocol detected higher infection rates by qPCR as compared to microscopy (Robinson et al., 2015; Hofmann et al., 2017). The qPCR assay exhibited very low sensitivity when light microscopy was used as a reference method, in particular when conventional DNA extraction was used. This is most likely due to the qPCR method needing further optimization. However, false positive light microscopy reads and loss of oocysts and sporozoites while transferring the dissected midguts or salivary glands from the glass slides to the tubes for heating or DNA extraction may also have played a role.

## Conclusions

In summary, we show that a qPCR assay can be used to detect very low numbers of mosquito stage *P. vivax* parasites. Furthermore, we show that by heating the mosquito guts and the head and thorax we save on costs and reduce the time taken to process the samples. We believe that this high-through-put setup will be a valuable tool in evaluating potential transmission blocking vaccines or antimalarials or for evaluating the infection status of field caught mosquitoes.

## Data availability statement

The raw data supporting the conclusions of this article will be made available by the authors, without undue reservation.

## Ethics statement

The studies involving human participants were reviewed and approved by Medical Research Advisory Committee of Papua New Guinea. Written informed consent to participate in this study was provided by the participants’ legal guardian/next of kin.

## Author contributions

Designed the study: LT and SK; Conducted the laboratory work: LT and EJ; Drafting and preparation of the manuscript: LT and SK; Critically revising the manuscript: SK, TB, EJ, and MK. All authors contributed to the article and approved the submitted version.
