# Supplementary material for: Using qPCR to compare the detection of Plasmodium vivax oocysts and sporozoites in Anopheles farauti mosquitoes between two DNA extraction methods
Source: Front Parasitol. 2023 Mar 16;2:1063452. doi: 10.3389/fpara.2023.1063452 (PMC11731789; doi:10.3389/fpara.2023.1063452)
Supplement: Supplementary file 1 [file DataSheet_1.pdf]

Supplementary 1

Table S1. Oocyst counts and whether they were processed by heating or DNA extraction.

Number of oocyst/mosquito	Number of Mosquitoes	Heating / DNA extraction
1	17	Heating
2	5	Heating
3	6	Heating
4	4	Heating
5	2	Heating
6	1	Heating
7	1	Heating
10	1	Heating
Pools	36	Heating
1	17	DNA extraction
2	4	DNA extraction
3	4	DNA extraction
4	5	DNA extraction
6	1	DNA extraction
8	1	DNA extraction
13	1	DNA extraction
14	1	DNA extraction
46	1	DNA extraction
Pools	36	DNA extraction

Table S2. Sporozoites classification and whether they were processed by heating or DNA extraction.

Sporozoite classification*	Number of Mosquitoes	Heating / DNA extraction
High	17	Heating
Moderate	9	Heating
Low	4	Heating
Negative	8	Heating
High	17	DNA extraction
Moderate	9	DNA extraction

Low	4	DNA extraction
Negative	8	DNA extraction

* High >100 sporozoites, Moderate 20 -100, Low 1-20 sporozoites
