# Supplementary material for: Using qPCR to compare the detection of Plasmodium vivax oocysts and sporozoites in Anopheles farauti mosquitoes between two DNA extraction methods
Source: Front Parasitol. 2023 Mar 16;2:1063452. doi: 10.3389/fpara.2023.1063452 (PMC11731789; doi:10.3389/fpara.2023.1063452)
Supplement: Supplementary file 2 [file DataSheet_2.pdf]

**Supplementary 2**

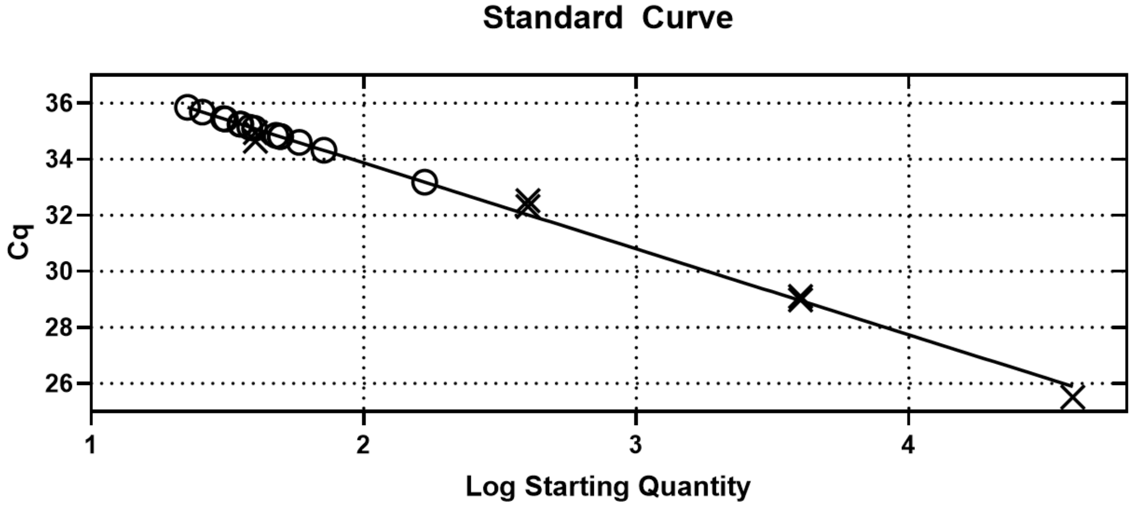


**Figure S1** Standard curve derived from known concentrations of plasmids. The plasmid concentrations ($10, 10^2, 10^3$ and $10^4$) are represented by the clear circles while the samples are represented by the cross.
