# Supplementary material for: Using qPCR to compare the detection of Plasmodium vivax oocysts and sporozoites in Anopheles farauti mosquitoes between two DNA extraction methods
Source: Front Parasitol. 2023 Mar 16;2:1063452. doi: 10.3389/fpara.2023.1063452 (PMC11731789; doi:10.3389/fpara.2023.1063452)
Supplement: Supplementary file 3 [file DataSheet_3.pdf]

Supplementary 3

Table S1. Primer sequences of for the qPCR assay to detect *P. falciparum* and *P. vivax* parasites

Species	Primer[1]	Sequence (5' - 3')
P. falcipaurm	**Pf_fwd**	TATTGCTTTTGAGAGGTTTTGTTACTTTG
	**Pf_rev**	ACCTCTGACATCTGAATACGAATGC
P. vivax	**Pv_fwd**	GCTTTGTAATTGGAATGATGGGAAT
	**Pv_rev**	ATGCGCACAAAGTCGATACGAAG

Table S2. Probe sequences for the qPCR assay to detect *P. falciparum* and *P. vivax* parasites.

Species	Probe[2]	Sequence (5' - 3')
P. falciparum	**Pf probe**	**6FAM**-ACGGGTAGTCATGATTGAGTT-**MGBNFQ**
P. vivax	**Pv probe**	**VIC**-AGCAACGCTTCTAGCTTA -**MGBNFQ**

Table S3. The reaction mix for the qPCR

qPCR Reaction mix
**Total volume** 14µL
2X Roche Master mix[1]
350nM per primer (forward and reverse)[2]
350nM per probe (forward and reverse)[3]
4µL of DNA

Table S4. The cycling conditions for the qPCR.

Thermo profile			
**Hold**	50°C	2min	
**Hold**	95°C	15min	
**Denaturation**	95°C	15sec	X 45
**Annealing**	60°C	1min	

1. LifeScience Roche, NSW, Australia
2. Integrated DNA Technologies (IDT), New Zealand
3. TaqMan MGB Probes ThermoFisher Scientific, Auckland, New Zealand
